# Noninvasive Early Detection and Recurrence Monitoring for Non‐Muscle‐Invasive Bladder Cancer via Urine Tumor DNA: A Prospective Clinical Study

**DOI:** 10.1002/mco2.70592

**Published:** 2026-01-15

**Authors:** Junlong Wu, Shengming Jin, Qianming Bai, Huina Wang, Huanqing Cheng, Xiaoyan Zhou, Yijun Shen, Chunguang Ma, Chengyuan Gu, Hui Chen, Yafeng Zhang, Libin Chen, Shahrokh F. Shariat, Feng Lou, Shanbo Cao, Yiping Zhu, Dingwei Ye

**Affiliations:** ^1^ Department of Urology Fudan University Shanghai Cancer Center Shanghai China; ^2^ Department of Oncology Shanghai Medical College Fudan University Shanghai China; ^3^ Department of Pathology Fudan University Shanghai Cancer Center Shanghai China; ^4^ Institute of Pathology Fudan University Shanghai China; ^5^ Acornmed Biotechnology Co., Ltd Beijing China; ^6^ Department of Urology Harbin Medical University Cancer Hospital Harbin China; ^7^ Department of Urology Comprehensive Cancer Center Medical University Vienna Vienna Austria; ^8^ Institute For Urology and Reproductive Health Sechenov University Moscow Russia; ^9^ Department of Urology University of Texas Southwestern Medical Center Dallas Texas USA; ^10^ Department of Urology Weill Cornell Medical College New York New York USA; ^11^ Division of Urology Department of Special Surgery University of Jordan Amman Jordan; ^12^ Research Center for Evidence Medicine Urology Department Tabriz University of Medical Sciences Tabriz Iran

**Keywords:** early detection, minimal residual disease, non‐muscle‐invasive bladder cancer, recurrence monitoring, urine tumor DNA

## Abstract

Conventional approaches for the detection and surveillance of non‐muscle invasive bladder cancer (NMIBC) remain invasive, burdensome, and costly. The utLIFE‐UC assay, designed to identify mutations and large copy number variations in urine, has demonstrated high accuracy in detecting urothelial carcinoma. Here, we assessed its efficacy in early detection of NMIBC, identifying minimal residual disease, and monitoring recurrence. Among 108 consecutive NMIBC patients evaluated, utLIFE‐UC exhibited a sensitivity of 90.5% in diagnosing NMIBC, with comparable performance in detecting both de novo and recurrent NMIBC. For patients undergoing repeat transurethral resection of bladder tumor (Re‐TURBT), the assay accurately identified all cases with residual tumor, achieving a 100% negative predictive value. Positive postoperative utLIFE‐UC results before the first follow‐up cystoscopy predicted a higher risk of future relapse. A positive test result at any time following TURBT was correlated with poorer recurrence‐free survival, whereas sustained negative test results indicated recurrence‐free status. Moreover, utLIFE‐UC could predict recurrence with a median lead time of 73.5 days prior to clinical confirmation. As the first prospective, longitudinal analysis of urinary tumor DNA in NMIBC, this study highlights the potential of utLIFE‐UC to enable earlier recurrence detection and improve risk stratification, potentially obviating unnecessary Re‐TURBT and surveillance cystoscopies.

## Graphical abstract

In NMIBC surveillance, positive postoperative utLIFE‐UC results before initial follow‐up cystoscopy were significantly associated with increased relapse risk. Longitudinal analysis showed that positive utLIFE‐UC results at any time after TURBT correlated with worse recurrence‐free survival, while sustained negative results predicted recurrence‐free status. This approach offers potential to substantially reduce dependence on cystoscopy, thereby revolutionizing NMIBC management and improving patient outcomes.

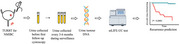

## 1 Introduction

Bladder cancer poses a substantial global health challenge, with an annual incidence exceeding 614,298 new cases worldwide [1]. This malignancy exerts a considerable economic burden on healthcare systems, largely attributable to the high costs associated with diagnostic procedures, therapeutic interventions, and long‐term disease management. Furthermore, the disease's inherent heterogeneity introduces additional complexities, as diverse tumor biology and variable patient responses to therapy complicate treatment optimization, highlighting the urgent need for personalized approaches.

Approximately 75% of newly diagnosed bladder cancer cases present with non‐muscle invasive bladder cancer (NMIBC) [2, 3], and these tumors are typically treated with transurethral resection of bladder tumor (TURBT), with or without adjunctive intravesical therapy. Despite receiving optimal treatment, a significant proportion of patients experience disease recurrence, with a 1‐year recurrence rate of 15%–61%, and a 5‐year recurrence rate of 31%–78% [4]. These recurrences may stem from either residual disease initially unrecognized or new tumor development due to field cancerization effects. Early identification of individuals at high risk for disease recurrence is crucial for informing therapeutic decision‐making and enhancing clinical outcomes.

Surveillance for NMIBC currently depends on frequent and long‐term cystoscopic examinations, often accompanied by urine cytology. This approach not only imposes substantial financial burdens but also causes considerable patient discomfort, adversely affecting quality of life [5, 6]. These challenges have created an urgent demand for the development of effective and non‐invasive methods that can accurately detect tumors, minimize unnecessary interventions, and facilitate early identification of disease recurrence.

Although numerous urine‐based biomarkers have been explored as potential tools for NMIBC monitoring, aiming to reduce the frequency of invasive cystoscopies, only a limited number have been approved by the Food and Drug Administration (FDA) [7]. Notably, none of these markers have been widely adopted in routine clinical practice, primarily due to their inherent limitations and insufficient clinical utility [8]. Among the FDA‐approved urine‐based biomarker assays for bladder cancer detection and surveillance, including the Bladder Tumor Antigen (BTA) test, Nuclear Matrix Protein 22 (NMP22) test, and UroVysion test, the reported diagnostic performance remains suboptimal, with an overall sensitivity of 57%–82% and a negative predictive value (NPV) of 21%–48% [9, 10].

The utLIFE‐UC is a novel urinary assay that integrates the detection of both genetic alterations and large copy number variants (CNVs). Our preliminary investigation demonstrated the clinical utility of utLIFE‐UC in identifying minimal residual disease (MRD) following neo‐adjuvant chemotherapy in patients with muscle‐invasive bladder cancer (MIBC) [11]. Based on these findings, we postulate that utLIFE‐UC may exhibit good diagnostic performance in identifying MRD after TURBT and monitoring disease recurrence during post‐treatment surveillance. To validate this hypothesis, we performed a prospective cohort study.

## 2 Results

### 2.1 Study Cohort

**FIGURE 1:**
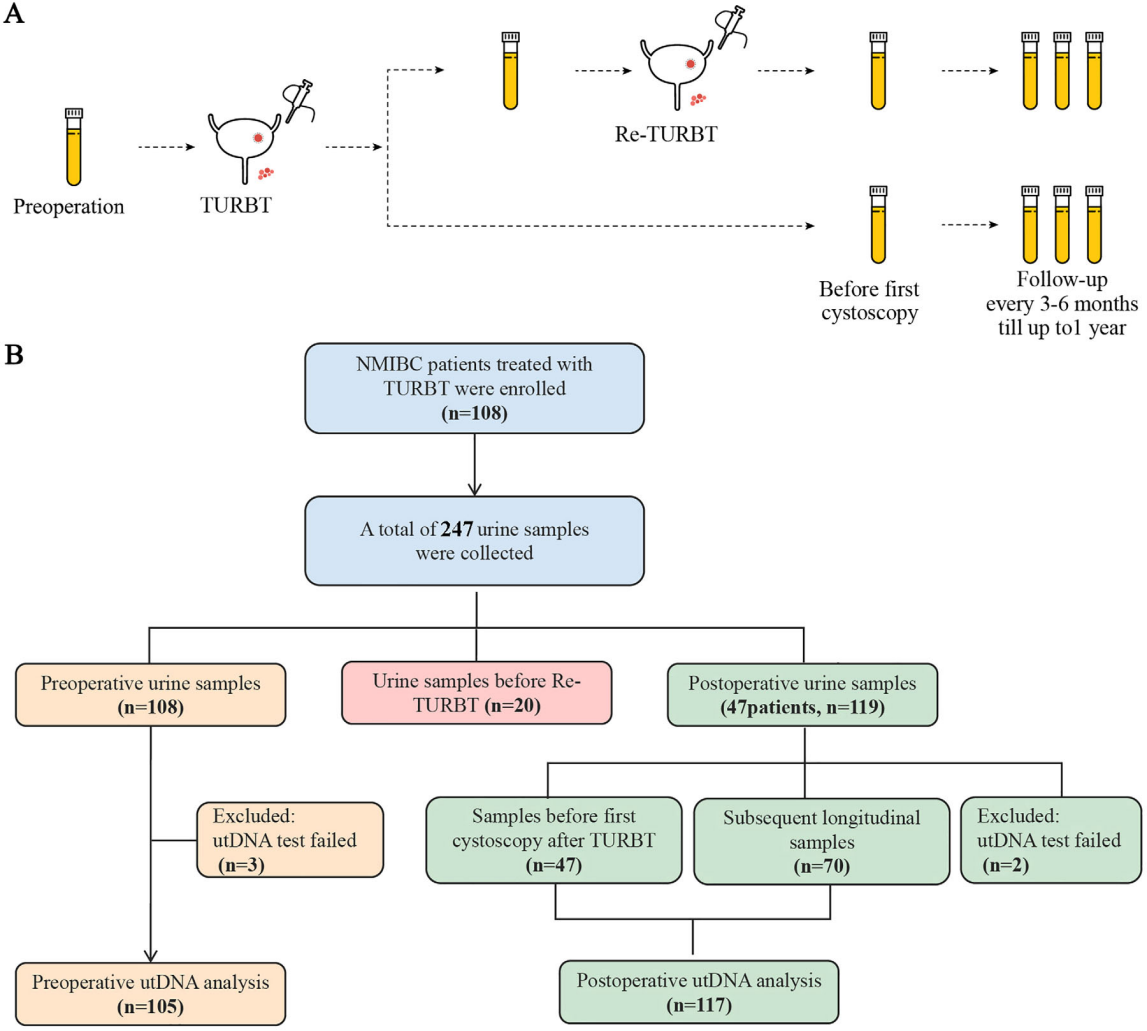
Study design and patient enrollment. (**A)** The overview of the urine collection time points. Urine samples were collected prior to transurethral resection of bladder tumor (TURBT) and repeat TURBT (Re‐TURBT) when indicated. Post‐surgical samples were collected every 3–6 months prior to patients undergoing routine cystoscopy. (**B)** A total of 247 urine samples from 108 patients were included for the analysis. The overview of samples available for each urinary tumor DNA (utDNA) analysis is displayed. NMIBC, non‐muscle invasive bladder cancer.

A flow diagram summarizing the study design is depicted in Figure 1. A total of 108 consecutive patients with histologically confirmed NMIBC were prospectively enrolled and categorized into two groups: A diagnostic test setting and a recurrence monitoring test setting. In the diagnostic test setting, all enrolled patients (*n* = 108) underwent preoperative utDNA testing; however, testing failed in three patients, excluding them from further analysis. For recurrence monitoring, while all patients received standard TURBT, only 47 patients met the inclusion criteria of having both postoperative urine specimens and complete follow‐up data. These patients contributed 117 evaluable samples for MRD assessment, yielding a mean of 2.5 tests per patient (ranging from 1 to 4). The clinical characteristics of these two settings are summarized in Table S1. In the diagnostic setting, the proportion of Ta and T1 tumors was 47.6% and 46.7%, respectively, while low‐grade (LG) and high‐grade (HG) tumors accounted for 27.6% and 67.6%, respectively. In the recurrence monitoring setting, the distribution of Ta and T1 tumors was 53.2% and 46.8%, respectively, with LG and HG tumors representing 21.3% and 76.6%, respectively.

### 2.2 Diagnostic Performance of utLIFE‐UC for Primary and Recurrent NMIBC Detection

**FIGURE 2:**
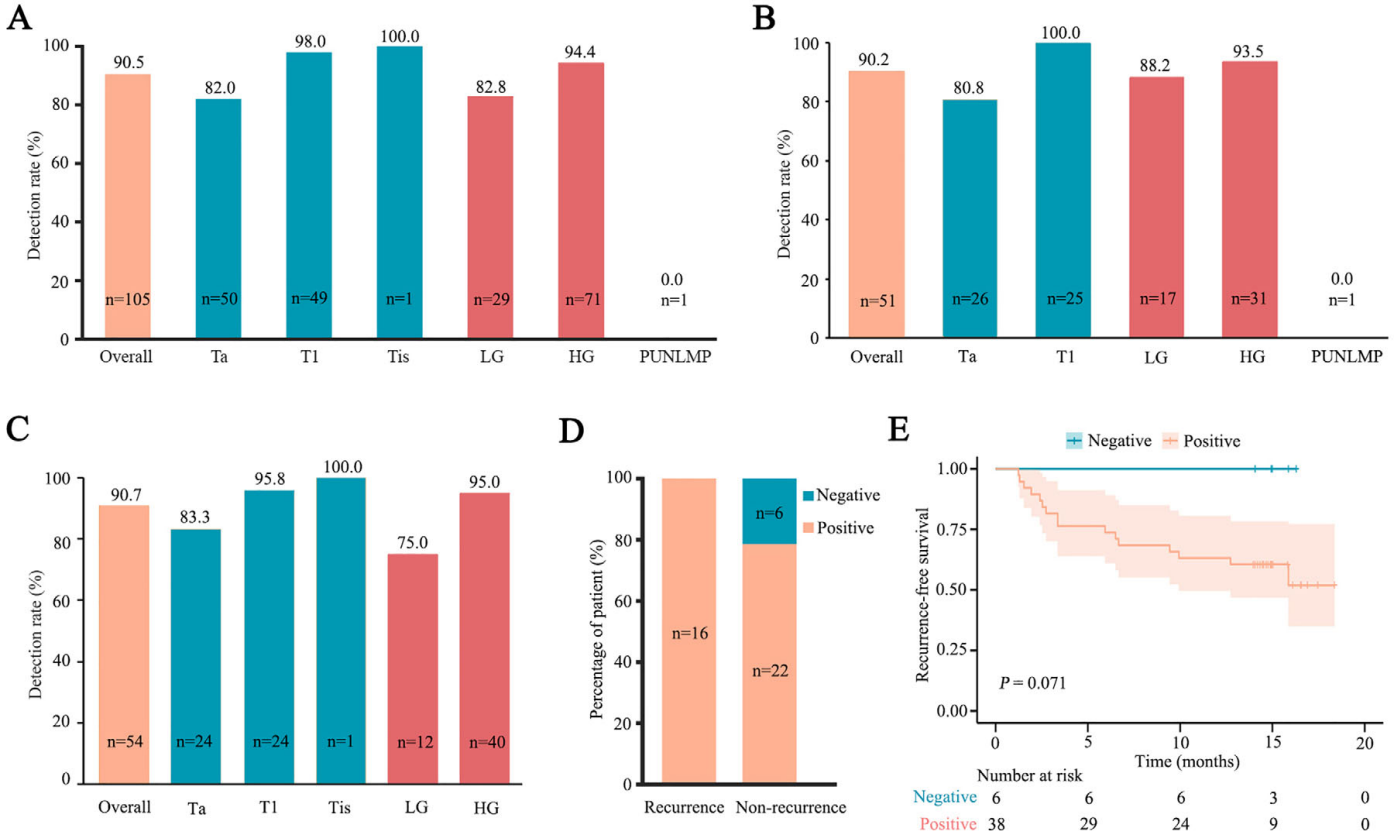
Preoperative detection of utDNA in NMIBC and its association with recurrence. (**A)** Detection rate of preoperative utDNA in total NMIBC patients with different T stage and tumor grade. (**B)** Detection rate of preoperative utDNA in newly diagnosed NMIBC patients with different T stage and tumor grade. (**C)** Detection rate of preoperative utDNA in recurrent NMIBC patients with different T stage and tumor grade. (**D)** The association between preoperative utDNA status and recurrence. (**E)** Kaplan–Meier estimates of recurrence‐free survival (RFS) for patients stratified by preoperative utDNA status. LG, low grade tumor; HG, high grade tumor; PUNLMP, papillary urothelial neoplasm of low malignant potential.

Among the 105 NMIBC patients with successfully evaluated preoperative samples, 95 (90.5%; 95% confidence interval (CI): 82.8%‐95.1%) tested positive on the utLIFE‐UC assay. The detection rates were 82.0% (95%CI: 68.1%‐90.9%) for Ta tumors and 98.0% (95%CI: 87.8%‐99.9%) for T1 tumors. The assay demonstrated a sensitivity of 82.8% (95%CI: 63.5%‐93.5%) for LG tumors and 94.4% (95%CI: 85.5%‐98.2%) for HG tumors (Figure 2A). No significant variations in detection rates were observed based on patient sex, age, or tumor number (Figure S1). The cohort included 51 newly diagnosed (de novo) patients and 54 patients with recurrent disease, demonstrating comparable detection rates of 90.2% (95% CI: 77.8%–96.3%) and 90.7% (95% CI: 78.9%–96.5%) (Figure 2B,C), respectively. Among de novo patients, detection rates were 80.8% (95% CI: 60.0%–92.7%) for Ta tumors versus 100% (95% CI: 83.4%–100.0%) for T1 tumors, and 88.2% (95% CI: 62.3%–97.9%) for LG versus 93.5% (95% CI: 77.2%–98.9%) for HG tumors (Figure 2B). In recurrent cases, corresponding rates were 83.3% (95% CI: 61.8%–94.5%) for Ta, 95.8% (95% CI: 76.9%–99.8%) for T1, 75.0% (95% CI: 42.8%–93.3%) for LG, and 95.0% (95% CI: 81.8%–99.1%) for HG tumors (Figure 2C).

Of the 47 NMIBC patients undergoing recurrence monitoring after TURBT, 16 (34.0%) developed disease recurrence during follow‐up (median duration: 14.5 months; interquartile range [IQR]: 8.1–15.1 months), while 31 (66.0%) remained recurrence‐free and continued active surveillance. Preoperative samples were successfully analyzed in 44 cases (93.6% of the monitoring cohort). All 16 recurrent patients showed positive preoperative utLIFE‐UC results. Among 28 non‐recurrent patients, 6 (21.4%) tested negative (Figure 2D), comprising 4 Ta tumors and 1 PUNLMP. Notably, patients with negative preoperative utLIFE‐UC results showed a trend toward better prognosis compared to positive cases (*p* = 0.071) (Figure 2E).

### 2.3 Utility of utLIFE‐UC to Identify Residual Tumor After Initial TURBT

**FIGURE 3:**
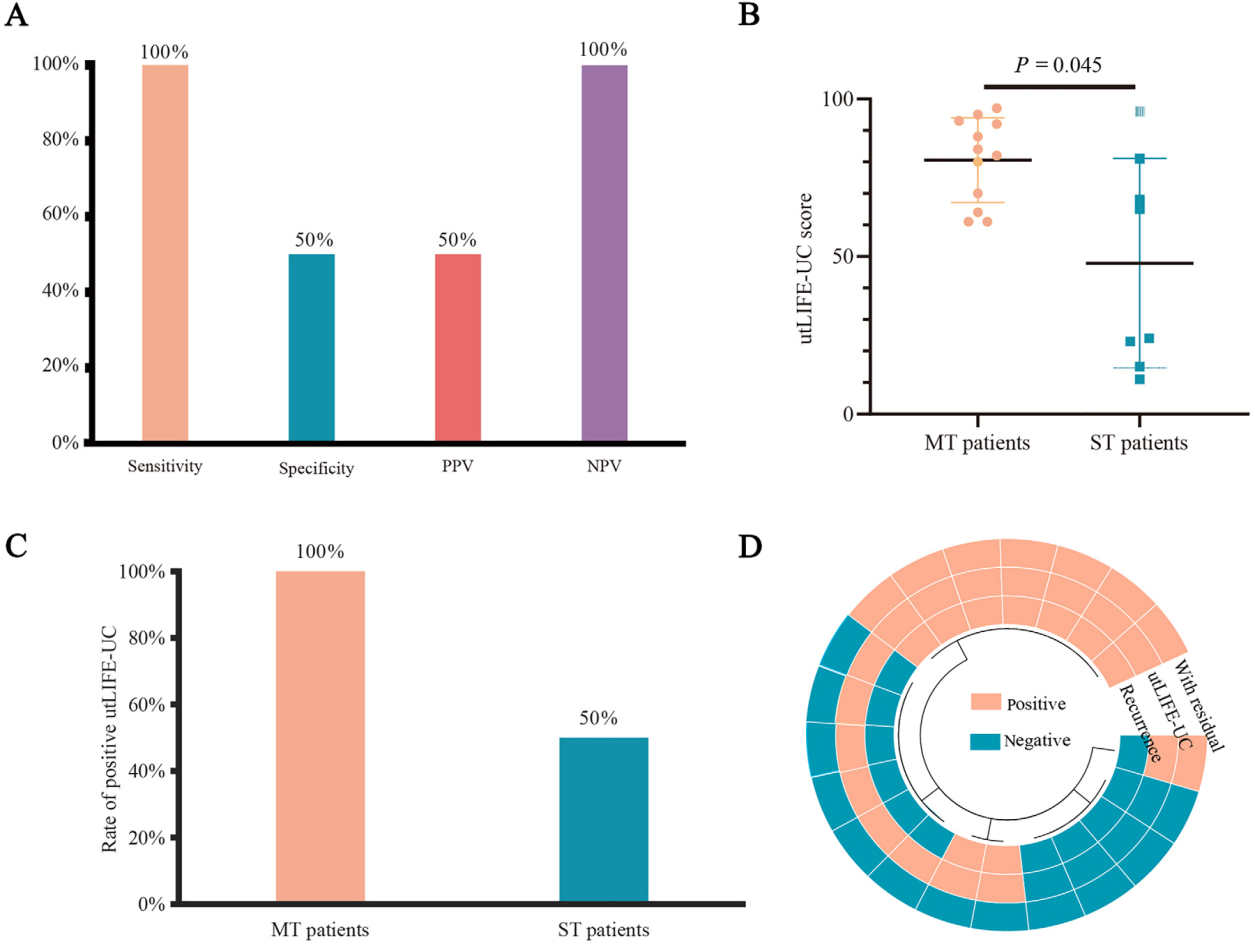
The performance of utLIFE‐UC in identifying residual disease after initial TURBT. (**A)** The sensitivity and negative predictive value (NPV) of utLIFE‐UC in identifying residual disease after initial TURBT. **(B)** Comparison of the utLIFE‐UC score between patients with multiple tumors (MT) and patients with single tumor (ST). (**C)** Rate of positive utLIFE‐UC test results before Re‐TURBT in MT patients and ST patients. (**D)** The association among residual disease status after initial TURBT, utLIFE‐UC test status before Re‐TURBT, and recurrence.

Twenty patients successfully underwent utDNA examination prior to Re‐TURBT to investigate the value of utLIFE‐UC in detecting residual tumors following initial TURBT. The tumor residual rate after initial TURBT was 40% (8/20). The utLIFE‐UC accurately identified 8 out of 8 (100%) cases with residual tumors, achieving a NPV of 100% (Figure 3A). Additional analysis revealed that the utLIFE‐UC score in patients with multiple tumors (MT) was higher than that in patients with a single tumor (ST) (*p =* 0.045) (Figure 3B). The utLIFE‐UC assay demonstrated a 100% positivity rate in MT patients following initial TURBT, compared to 50% in ST patients (Figure 3C). Among the 20 patients analyzed, disease relapse occurred in nine cases during the follow‐up period. Of particular clinical significance, two patients with positive utLIFE‐UC results despite the absence of residual tumors subsequently experienced disease relapse, including one case with rapid recurrence (recurrence‐free survival [RFS] = 2.5 months). Notably, all patients with negative utLIFE‐UC results prior to Re‐TURBT maintained disease‐free status throughout the observation period (Figure 3D).

### 2.4 Early Recurrence Detection by utLIFE‐UC Before Initial Post‐TURBT Cystoscopy

**FIGURE 4:**
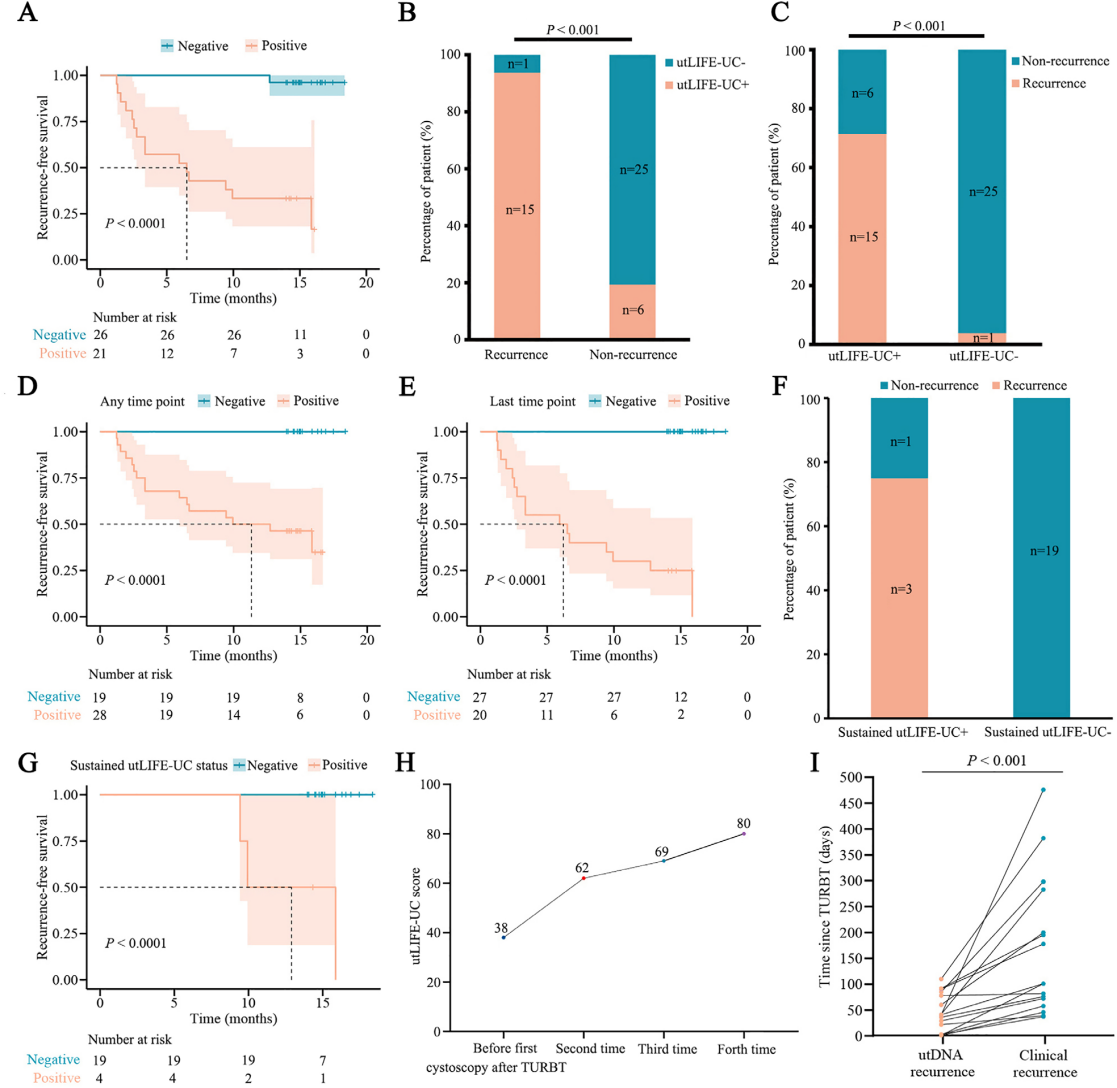
Postoperative utDNA analysis for detecting NMIBC. (**A)** Kaplan–Meier estimates of RFS for patients stratified by utLIFE‐UC test status before first cystoscopy after TURBT. (**B, C)** The association between utLIFE‐UC test status before first cystoscopy after TURBT and recurrence during the follow‐up period. (**D)** Kaplan–Meier estimates of RFS for patients stratified by utLIFE‐UC test status at any time point after TURBT. (**E)** Kaplan–Meier estimates of RFS for patients stratified by utLIFE‐UC test status at last time point after TURBT. (**F)** The correlation between sustained utLIFE‐UC test status after TURBT and recurrence during the follow‐up period. (**G)** Kaplan–Meier estimates of RFS for patients stratified by sustained utLIFE‐UC test status after TURBT. (**H)** Example of a patient's changeable utLIFE‐UC test status during surveillance. The patient's minimal tumor was early detected by utLIFE‐UC, but was missed by cystoscopy. (**I)** The lead time of recurrence between utLIFE‐UC test results and clinical confirmed recurrence. utLIFE‐UC+, utLIFE‐UC positive; utLIFE‐UC‐, utLIFE‐UC negative.

To further evaluate postoperative MRD and stratify patients’ risk of recurrence, we performed utLIFE‐UC analysis on urine samples collected prior to the first follow‐up cystoscopy after TURBT. Among the 47 patients with available urine samples, 21 (44.7%) tested positive, and 26 (55.3%) tested negative for utLIFE‐UC. Patients with positive utLIFE‐UC results demonstrated significantly higher recurrence risk compared to their negative counterparts (hazard ratio [HR], 29.86; 95% CI, 10.57–84.35; *p* < 0.0001; median RFS, 6.5 months vs. unreached) (Figure 4A). At the end of the follow‐up period, utLIFE‐UC positivity was observed in 93.8% (15/16) relapsed patients, compared to only 19.4% (6/31) in the recurrence‐free group (*p* < 0.001) (Figure 4B). Among the 26 utLIFE‐UC negative patients, 25 remained recurrence‐free, yielding a NPV of 96.2% (95%CI: 78.4%–99.8%). Conversely, of the 21 utLIFE‐UC positive patients, 15 experienced relapse, resulting in a positive predictive value (PPV) of 71.4% (95%CI: 47.7%–87.8%) (Figure 4C). The clinical utility of the utLIFE‐UC assay was assessed in a table summarizing the estimated number of avoided cystoscopies (true negatives, TN), missed tumors (false negatives, FN), and correctly identified cancer cases (true positives, TP). These figures were also extrapolated to a cohort of 1000 patients, assuming a 20% recurrence rate (Table S2).

### 2.5 Longitudinal utLIFE‐UC Assessment for Recurrence Prediction

**FIGURE 5:**
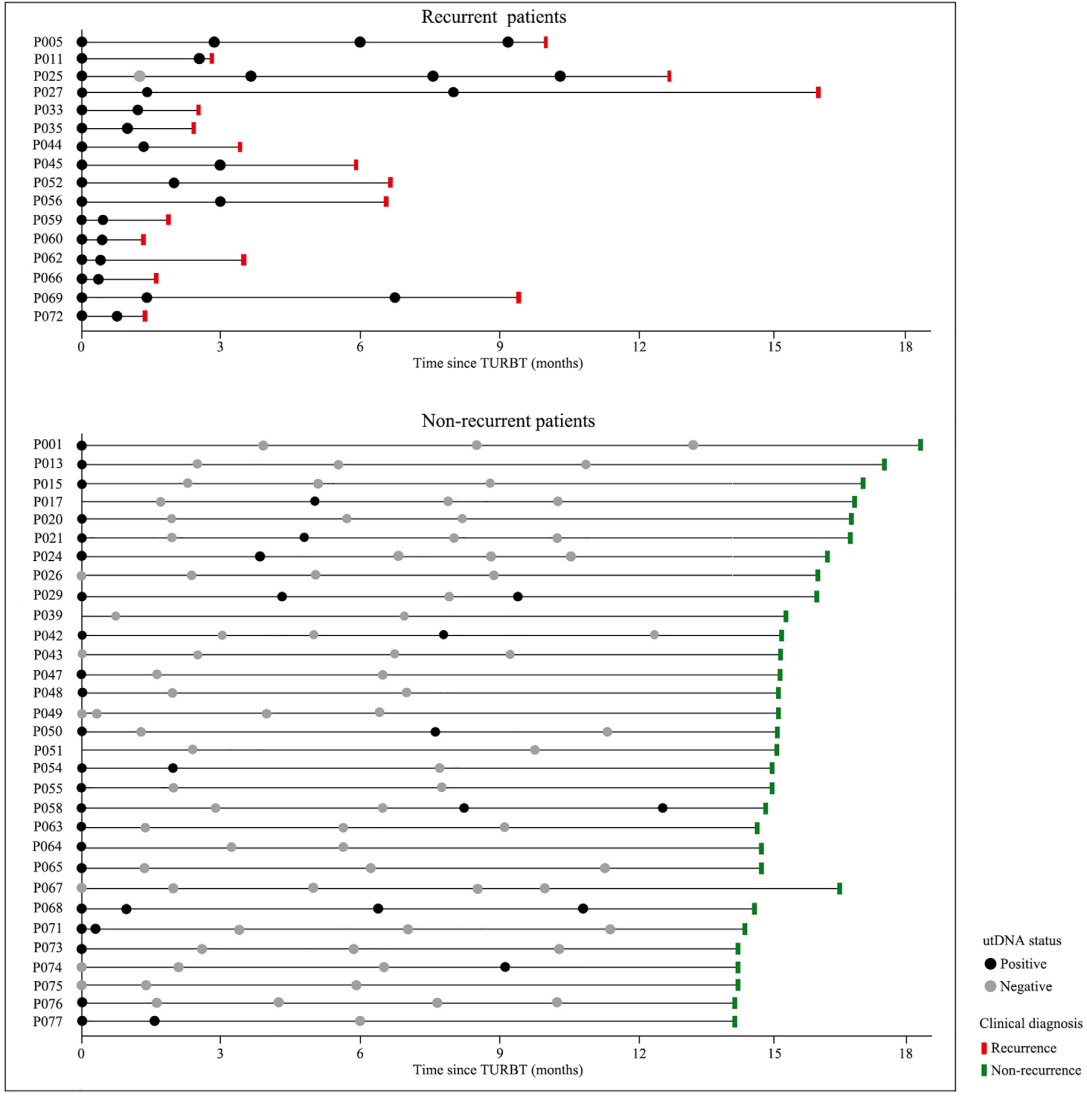
An overview of utLIFE‐UC results and disease course for patients in the longitudinal utDNA analysis.

To evaluate the potential of longitudinal utLIFE‐UC analysis as a dynamic biomarker for recurrence prediction during post‐surgical surveillance, we conducted a comprehensive analysis of 16 recurrent and 31 non‐recurrent patients, with detailed disease course and urine collection timelines illustrated in Figure 5. Among the 47 monitored patients, 28 (59.5%) showed positive utLIFE‐UC results across 38 samples. Longitudinal analysis revealed that positive utLIFE‐UC results at any time point were significantly associated with reduced RFS (*p* < 0.0001) (Figure 4D). Further analysis focusing on the final sampling results demonstrated that patients with positive utLIFE‐UC outcomes maintained significantly worse RFS compared to negative cases (*p* < 0.0001) (Figure 4E). Notably, among 23 patients with consistent longitudinal utLIFE‐UC results, 75.0% (3/4) of those with sustained positivity experienced relapse, while none of the 19 patients with persistent negative results (0%) developed recurrence (Figure 4F). Patients maintaining negative utLIFE‐UC status showed significantly prolonged RFS compared to their positive counterparts (*p* < 0.0001) (Figure 4G), underscoring the prognostic value of sustained negativity.

The clinical utility of longitudinal utLIFE‐UC monitoring was further exemplified by a representative case demonstrating dynamic test results during surveillance. Initial negative utLIFE‐UC results preceding the first post‐TURBT cystoscopy were followed by three consecutive positive results, despite negative findings in the first three cystoscopies. Clinical recurrence was subsequently confirmed three months after the fourth test (Figure 4H). Importantly, in recurrent patients with positive utLIFE‐UC results, the test was able to predict recurrence with a median lead time of 73.5 days (IQR, 42.3–158.3 days; *p* < 0.001) prior to clinical recurrence detection (Figure 4I).

### 2.6 Redefined Risk Stratification in Clinically Intermediate‐ and High‐Risk NMIBC

**FIGURE 6:**
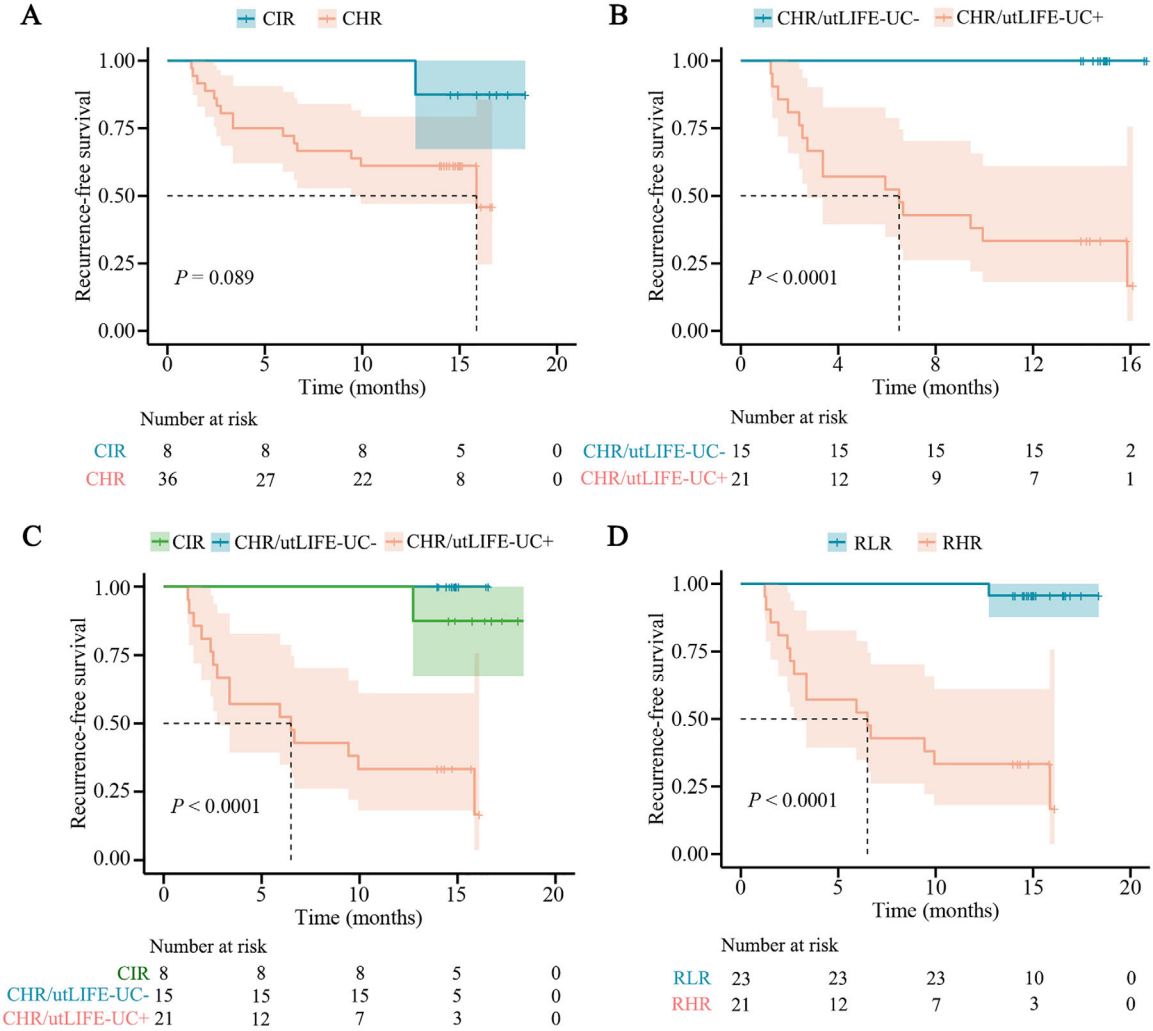
Risk stratification refinement in clinically intermediate‐ and high‐risk NMIBC using utLIFE‐UC testing. (**A)** Kaplan–Meier estimates of RFS between clinically intermediate‐ and high‐risk NMIBC patients. (**B)** Kaplan–Meier estimates of RFS in clinically high‐risk patients stratified by utLIFE‐UC status. (**C)** Kaplan–Meier estimates of RFS for clinically intermediate‐risk patients and clinically high‐risk patients stratified by utLIFE‐UC test status. (**D)** Kaplan–Meier estimates of RFS according to the redefined risk classification incorporating both clinical risk and utLIFE‐UC status. CIR, clinically intermediate‐risk; CHR, clinically high‐risk; CHR/utLIFE‐UC‐, clinically high‐risk/utLIFE‐UC negative; CHR/utLIFE‐UC+, clinically high‐risk/utLIFE‐UC positive; RLR, redefined low risk; RHR, redefined high risk.

Subsequently, we further investigated the utility of postoperative utLIFE‐UC testing in refining recurrence risk stratification within clinically defined risk subgroups (low‐, intermediate‐, and high‐risk). Given the absence of recurrence in the low‐risk cohort during follow‐up, our analysis focused exclusively on the intermediate‐ and high‐risk patients. Notably, no significant difference in RFS was observed between these two groups (Figure 6A), prompting us to combine them for subsequent prognostic evaluation. We then examined whether utLIFE‐UC testing could improve risk stratification in intermediate‐ and high‐risk NMIBC.

In high‐risk NMIBC cases, those with postoperative negative utLIFE‐UC results (tested prior to initial post‐TURBT cystoscopy) demonstrated significantly better RFS compared to positive cases (*p* < 0.0001; Figure 6B). Notably, all intermediate‐risk patients tested negative for utLIFE‐UC and maintained a 1‐year RFS rate of 100%. When incorporating utLIFE‐UC testing into risk stratification, we observed that high‐risk patients with positive utLIFE‐UC results had the worst outcomes, exhibiting only a 33.3% 1‐year RFS rate. In contrast, both intermediate‐risk patients and high‐risk patients with negative utLIFE‐UC results achieved a 1‐year RFS rate of 100% (Figure 6C). These findings supported a modified risk classification system for intermediate‐ and high‐risk NMIBC, in which patients with positive utLIFE‐UC results were categorized as redefined high‐risk, while all others were classified as redefined low‐risk. Application of these novel stratification criteria through Kaplan–Meier analysis showed remarkable discrimination of relapse risk (HR = 26.49; 95% CI: 9.55–73.45; *p* < 0.0001) (Figure 6D). During surveillance, the redefined high‐risk group exhibited a 71.4% recurrence rate, compared to just 4.3% in the refined low‐risk group. Of particular clinical importance, longitudinal utLIFE‐UC monitoring revealed that patients maintaining consistently negative results remained recurrence‐free, irrespective of their redefined risk category, further underscoring the strong prognostic value of sustained utLIFE‐UC negativity.

### 2.7 Potential Clinical Utility Mode of utLIFE‐UC in NMIBC Recurrence Monitoring

**FIGURE 7:**
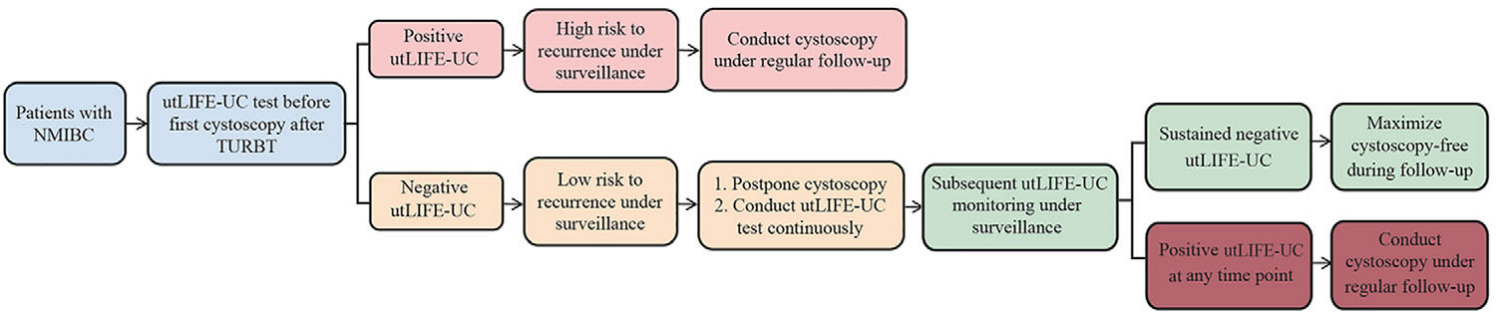
The potential clinical application pattern of utLIFE‐UC for NMIBC recurrence monitoring.

Based on the aforementioned findings, we propose a potential clinical application pattern to incorporate utLIFE‐UC into clinical practice for monitoring NMIBC recurrence (Figure 7). The utDNA status of preoperative samples aids in predicting prognosis. During post‐TURBT surveillance, utLIFE‐UC results obtained prior to the first follow‐up cystoscopy serve as critical indicators for clinical decision‐making. For patients with positive utLIFE‐UC results at this time point, the elevated recurrence risk necessitates confirmed cystoscopy as part of regular follow‐up protocols. Conversely, negative utLIFE‐UC results indicate a low short‐term relapse risk, allowing for modification of surveillance strategies. In such cases, scheduled cystoscopies may be deferred, surveillance frequency reduced, and longitudinal utLIFE‐UC testing implemented as an alternative monitoring approach. During longitudinal utLIFE‐UC monitoring, patients maintaining consistent negative results may extend the interval between cystoscopic examinations. However, the detection of positive utLIFE‐UC results at any monitoring time point warrants implementation of confirmed cystoscopy within the regular follow‐up protocol.

## 3 Discussion

To our knowledge, this represents the first prospective study focusing on longitudinal utDNA measurements for MRD detection in NMIBC, demonstrating a strong correlation between MRD status and clinical outcomes. Our findings reveal that patients exhibiting positive MRD status prior to the initial post‐TURBT cystoscopy are at significantly higher risk of early recurrence. Conversely, patients maintaining persistent negative MRD status over time constitute a distinct subgroup with an extremely low risk of recurrence, potentially warranting adjustments in surveillance frequency.

Overall, the detection rate of preoperative positive utDNA in the entire population was 90.5%. The assay demonstrated high sensitivity in detecting both primary (90.2%) and recurrent (90.7%) NMIBC cases. These prospective findings confirm the efficacy of utLIFE‐UC as a noninvasive diagnostic tool for de novo and recurrent NMIBC in clinical practice, suggesting its potential utility in NMIBC recurrence monitoring.

Numerous studies have demonstrated that residual disease rates after initial TURBT vary significantly, ranging from 17% to 78% [12, 13, 14]. In addition, the positive rate for Re‐TURBT shows substantial variability and is strongly linked to the efficiency of the initial surgery [15], suggesting that some patients might be subjected to unnecessary Re‐TURBT. In the present study, the utLIFE‐UC accurately identified all patients with residual tumors. Moreover, the assay displayed a high NPV of 100%, and patients with no residual disease detected by utLIFE‐UC retained non‐recurrence during the follow‐up period, potentially qualifying for Re‐TURBT avoidance. Notably, the number of tumors significantly influenced utLIFE‐UC positivity rates after initial TURBT, with 100.0% and 50.0% detection rates in MT and ST patients, respectively. These findings position utLIFE‐UC as a valuable tool for stratifying ST patients requiring Re‐TURBT. Importantly, two utLIFE‐UC‐positive cases without detectable residual tumors subsequently experienced recurrence, underscoring the assay's superior sensitivity in identifying residual disease.

A considerable proportion of patients with NMIBC develop recurrence following TURBT, necessitating frequent and prolonged invasive cystoscopic surveillance. The development of effective noninvasive tools for early recurrence detection is therefore clinically imperative. While recent studies have investigated the potential use of postoperative utDNA for NMIBC surveillance [16, 17], their limitations in assessing utDNA at a single random time point and binary detection status have left the clinical utility of serial utDNA monitoring for dynamic recurrence risk prediction unresolved. Our study revealed that patients with positive utLIFE‐UC results prior to their first post‐TURBT surveillance cystoscopy demonstrated significantly higher recurrence risk, with a median RFS of merely 6.5 months. At this time point, utLIFE‐UC achieved 93.8% sensitivity and 96.2% NPV for recurrence detection, illustrating its efficacy and reliability in identifying MRD and forecasting recurrence. Given the test's limited PPV, cystoscopic confirmation remains essential for definitive diagnosis. Its strong NPV provides substantial clinical benefit for patients with negative results who can omit cystoscopy, as these results reliably indicate absence of recurrence. Longitudinal analysis further demonstrated that utLIFE‐UC positivity at any surveillance time point correlated with increased recurrence risk. Notably, patients maintaining persistent utLIFE‐UC negativity remained recurrence‐free throughout follow‐up, similar with observations in lung and colorectal cancers [18, 19]. These findings identify a distinct subgroup of patients with postoperative persistent utLIFE‐UC negativity who exhibit an extremely low risk of relapse, potentially qualifying for modified surveillance strategies incorporating extended cystoscopy intervals and longitudinal utLIFE‐UC monitoring.

Our study revealed a 1‐year recurrence rate of 31.8% (14/44) in intermediate‐ and high‐risk NMIBC patients, consistent with published rates of approximately 30% [20, 21], confirming the substantial recurrence risk in this population. These findings highlight the critical need for refined risk stratification strategies in NMIBC management. Notably, utLIFE‐UC testing prior to initial post‐TURBT cystoscopy significantly improved recurrence risk prediction accuracy, enabling development of a novel stratification framework. This approach identified a redefined low‐risk subgroup (including both intermediate‐risk patients and high‐risk patients with negative utLIFE‐UC results) that represented over 50% of traditional intermediate‐ and high‐risk cases and achieved 100% 1‐year RFS, comparable to clinical low‐risk NMIBC outcomes. By integrating clinical parameters with utLIFE‐UC molecular assessment, this study advances precision medicine in bladder cancer care through optimized risk stratification.

Some limitations of this study should be acknowledged. First, the relatively limited sample size and follow‐up data restrict comprehensive evaluation of utLIFE‐UC's stratification potential in clinically low‐risk patient populations. Second, the median follow‐up duration of 14.5 months may be insufficient for assessing long‐term clinical outcomes associated with postoperative utLIFE‐UC status. Third, urine cytology and fluorescence in situ hybridization (FISH) were not performed concurrently with utLIFE‐UC assay, precluding direct comparison of these methods for MRD detection. Finally, as the study was designed as a preliminary exploration of the model's diagnostic efficacy in NMIBC, it does not allow for the determination of specificity and NPV. Despite these limitations, our study establishes the clinical feasibility of utDNA‐based MRD detection for recurrence prediction during surveillance, which will be further validated through large‐scale prospective studies with extended follow‐up periods.

## 4 Conclusions

This study systematically evaluates the clinical utility of utDNA in bladder cancer management, with a focus on its applications in MRD detection, risk stratification, and recurrence surveillance. The analysis highlights the utDNA‐based assay utLIFE‐UC, which integrates detection of genetic alterations and CNVs, as a promising adjunctive tool to enhance monitoring accuracy. While current assay costs are comparable to cystoscopy and exceed urine cytology, the assay's high‐throughput analytical capacity and potential for further optimization suggest favorable long‐term cost‐effectiveness relative to conventional methods. A key advantage of this technology lies in reducing unnecessary cystoscopies during surveillance, thereby lowering patient burden and healthcare costs, though cystoscopic confirmation remains essential for definitive diagnosis. Collectively, these findings support the integration of utDNA monitoring into clinical practice to decrease reliance on invasive procedures, advance personalized management of NMIBC, and improve patient outcomes.

## 5 Materials and Methods

### 5.1 Patients and Study Design

Patients with NMIBC from December 2022 to June 2023 were prospectively recruited from Fudan University Shanghai Cancer Center and Harbin Medical University Cancer Hospital. This study was approved by the ethical committee of Fudan University Shanghai Cancer Center, and registered on Chinese Clinical Trial Registry (Trial No. ChiCTR2300072635). Written informed consent was obtained from all patients. Eligible participants were required to meet the following criteria: (1) Clinically confirmed NMIBC scheduled for TURBT; (2) age ≥ 18 years; (3) willing to provide morning urine samples. Participants will be excluded if they meet any of the following: (1) Disease progression from recurrent NMIBC to MIBC; (2) concurrent other genitourinary malignancies (including renal cell carcinoma or prostate cancer); (3) participation in other interventional clinical trials within 3 months prior to enrollment; (4) inadequate urine sample quantity/quality or technical failure of utLIFE‐UC assay. For the eligible participants, urine samples were collected before TURBT and repeat TURBT (Re‐TURBT) as needed. Post‐surgical follow‐up samples were collected every 3–6 months prior to patients undergoing routine cystoscopy. The specifics of the urine collection time points are illustrated in Figure 1A. utLIFE‐UC is an advanced urinary diagnostic assay we previously developed, which employs a multidimensional bioinformatic approach to analyze urinary tumor DNA (utDNA), including somatic mutations and large CNVs. This method, as detailed in our earlier study [11], was employed to analyze the samples.

### 5.2 Urine Sample Processing

First‐void morning mid‐stream urine samples (approximately 100 mL) were collected from all participants. The urine supernatant was collected utilizing a urine DNA Storage Tube (CWBIO) for urinary cell‐free DNA (ucfDNA) extraction. Remaining urine sample was collected in a sterile tube containing urine conditioning buffer (UCB, ZYMO) for exfoliated cell DNA (uexDNA) extraction. All samples were transported to the laboratory within 72 h under conditions maintained at 2°C–8°C. Urine supernatants were centrifuged at 1600 × *g* and 16,000 × *g* for 10 min each. The urine sediment was centrifuged at 1600 × *g* for 10 min and subsequently stored at −80°C until DNA extraction. For library construction, a total of 1–30 ng of ucfDNA and 100 ng of uexDNA were used. Sequencing was conducted on Novaseq6000 platform (Illumina) in 150PE mode.

### 5.3 Mutation Analysis

Following targeted deep sequencing of ucfDNA, the low‐quality sequencing data were removed and the remaining sequencing reads were aligned to the hg19 version of the reference human genome with Burrows‐Wheeler Aligner (BWA, version 0.7.12). PCR duplicates were identified by MarkDuplicates tool in Picard. IndelRealigner and BaseRecalibrator tool in the Genome Analysis Toolkit (GATK, version 3.8) were used to realign and recalibrate the BWA alignment results. Sentieon TNhaplotyper was used to recognize somatic mutations. Nonsilent mutations included exonic nonsynonymous mutations, splicing mutations, stop codon mutations, frameshift insertion mutations, frameshift deletion mutations, nonframeshift insertion mutations, and nonframeshift deletion mutations. All silent mutations were excluded. Nonsilent mutations with a mutant allele frequency (MAF) < 0.01, reference base depth < 10, and allele base depth < 3 were filtered out.

### 5.4 Large CNV Analysis

Large CNV analysis was performed according to the shallow whole‐genome sequencing (1×WGS) data of uexDNA. The coverage was evaluated for every 200 k bin in all samples and was then corrected by GC context and self‐standardization. Normal samples acted as control to exclude telomeres, centromeres, and repetitive regions with significant noise or lacking genetic information. After standardization, calculations for all samples were performed using the following algorithm: N=∑segmentlength(|log2ratio|>Cutoff_1)

If the absolute value of the segment exceeds Cutoff_1, the segment is deemed to have CNV events.

### 5.5 Calculation of utLIFE‐UC Score

The utLIFE‐UC model integrated a comprehensive feature matrix that encompassed both somatic mutations and large CNVs. This matrix acts as the input for our classification model, which is fundamentally an SVM (Support Vector Machine) model. The raw output score of the utLIFE‐UC model spans continuously from 0 to 1, indicating the probability of urothelial carcinoma. The threshold for the model is established at 0.6, as determined by the maximum Youden Index value. To enhance comprehension for both medical professionals and patients, we have scaled this score by multiplying it by 100, thereby converting the range to a more intuitive scale of 0 to 100, and the threshold has been adjusted to 60. If the adjusted utLIFE‐UC score is equal to or below the threshold, the individual is classified as “negative”, indicating a lower likelihood of having urothelial carcinoma. Conversely, a score exceeding 60 is classified as “positive”, indicating the presence of urothelial carcinoma. This binary classification contributes to the clinical decision‐making process, providing a clear and actionable interpretation of the model's predictive output.

### 5.6 Statistical Analysis

Statistical analyses were performed using GraphPad Prism software (version 8.0.2) and R software (version 4.1.3). Fisher's exact test was conducted to analyze categorical data and Mann–Whitney test was conducted for continuous data analysis. Survival analyses were visualized with the Kaplan–Meier method and analyzed with the log‐rank test. A two‐sided *p* < 0.05 was considered as statistically significant.

## Author Contributions

D. Y., Y. Z., S. C., and F. L. designed the study. J. W., S. J., Q. B., H. W., H. Cheng., X. Z., Y. S., C. M., C. G., H. Chen, Y. Z., L. C., and S. F. S. analyzed and interpreted the data. J. W., S. J., Q. B., H. W., and H. Cheng wrote the article, which was edited and revised by all authors. All authors have read and approved the final manuscript.

## Funding

This work was supported by Beijing Xisike Clinical Oncology Research Foundation (grant number Y‐Young2023‐0087), Shanghai “Rising Stars of Medical Talents” Youth Development Program, and Shanghai Municipal Health Bureau Project (grant number 2020CXJQ03).

## Ethics Statement

This study was approved by the ethical committee of Fudan University Shanghai Cancer Center (approval number: 2210263‐3). Informed consent was obtained from all participants in the study.

## Conflicts of Interest

H.W., H.C., Y.Z., L.C., F.L., and S.C. are employees of Acornmed Biotechnology Co., Ltd. The other authors declare that they have no conflicts of interest.

## Supporting information




**Figure. S1**: Detection rate of preoperative utDNA in NMIBC patients with different tumor number, age, and sex.
**Table S1**: Clinical features of all the recruited NMIBC patients.
**Table S2**: Clinical utility assessment of the utLIFE‐UC assay.

## Data Availability

The datasets used and/or analyzed during the current study are available in Genome Sequence Archive under project PRJCA053939.
