# Supplementary material for: Noninvasive Early Detection and Recurrence Monitoring for Non‐Muscle‐Invasive Bladder Cancer via Urine Tumor DNA: A Prospective Clinical Study
Source: MedComm (2020). 2026 Jan 15;7(2):e70592. doi: 10.1002/mco2.70592 (PMC12805463; doi:10.1002/mco2.70592)
Supplement: Supplementary file 1 — Figure. S1: Detection rate of preoperative utDNA in NMIBC patients with different tumor number, age, and sex. Table S1: Clinical features of all the recruited NMIBC patients. Table S2: Clinical utility assessment of the utLIFE‐UC assay. [file MCO2-7-e70592-s001.docx]

Title: Noninvasive early detection and recurrence monitoring for non-muscle-invasive bladder cancer via urine tumor DNA: a prospective clinical study

**Running title:** Detection and surveillance of NMIBC using utDNA

**Author/institution**

Junlong Wu^1,2#^, Shengming Jin^1,2#^, Qianming Bai^2,3,4#^, Huina Wang^5#^, Huanqing Cheng^5#^, Xiaoyan Zhou^2,3,4^, Yijun Shen^1,2^, Chunguang Ma^1,2^, Chengyuan Gu^1,2^, Hui Chen^6^, Yafeng Zhang^5^, Libin Chen^5^, Shahrokh F Shariat^7,8,9,10,11,12^, Feng Lou^5,*^, Shanbo Cao^5,*^, Yiping Zhu^1,2,*^ and Dingwei Ye^1,2,*^

^1^Department of Urology, Fudan University Shanghai Cancer Center, Shanghai, China.

^2^Department of Oncology, Shanghai Medical College, Fudan University, Shanghai, China.

^3^Department of Pathology, Fudan University Shanghai Cancer Center, Shanghai, China.

^4^Institute of Pathology, Fudan University, Shanghai, China.

^5^Acornmed Biotechnology Co., Ltd, Beijing, China.

^6^Department of Urology, Harbin Medical University Cancer Hospital, Harbin, China.

^7^Department of Urology, Comprehensive Cancer Center Medical University Vienna, Vienna, Austria.

^8^Institute for Urology and Reproductive Health, Sechenov University, Moscow, Russia.

^9^Department of Urology, University of Texas Southwestern Medical Center, Dallas, TX, USA.

^10^Department of Urology, Weill Cornell Medical College, New York, NY, USA.

^11^Division of Urology, Department of Special Surgery, University of Jordan, Amman, Jordan.

^12^Research Center for Evidence Medicine, Urology Department Tabriz University of Medical Sciences, Tabriz, Iran.

***Corresponding author**

Dingwei Ye, Department of Urology, Fudan University Shanghai Cancer Center, Shanghai, China; Department of Oncology, Shanghai Medical College, Fudan University, Shanghai, China. E-mail: [dingwei_ye1963@163.com](mailto:dingwei_ye1963@163.com);

Yiping Zhu, Department of Urology, Fudan University Shanghai Cancer Center, Shanghai, China; Department of Oncology, Shanghai Medical College, Fudan University, Shanghai, China. E-mail: [qdzhuyiping@aliyun.com](mailto:qdzhuyiping@aliyun.com);

Shanbo Cao, Acornmed Biotechnology Co., Ltd, Beijing, China. E-mail: [shanbocao@acornmed.com](mailto:shanbocao@acornmed.com);

Feng Lou, Acornmed Biotechnology Co., Ltd, Beijing, China. E-mail: [fenglou@acornmed.com](mailto:fenglou@acornmed.com).

**^#^These authors contributed equally:** Junlong Wu, Shengming Jin, Qianming Bai, Huina Wang, Huanqing Cheng

**Supplementary Figures and Tables**

**
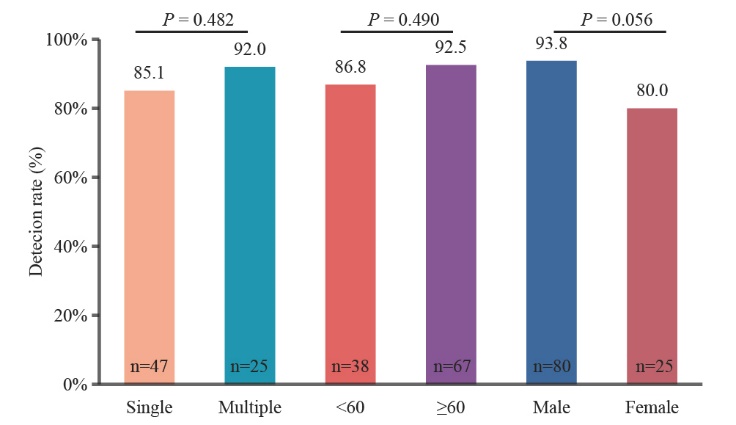
**

**Figure. S1** Detection rate of preoperative utDNA in NMIBC patients with different tumor number, age, and sex.

| Characteristics | NMIBC patients with  preoperative samples  (n = 105) | NMIBC with follow-up data and postoperative samples (n = 47) |
| --- | --- | --- |
| Sex |  |  |
| Female | 25 (23.8%) | 11 (23.4%) |
| Male | 80 (76.2%) | 36 (76.6%) |
| Age, median (range) | 65 (42-91) | 63 (46-81) |
| Smoking |  |  |
| Yes | 17 (16.2%) | 2 (4.3%) |
| No | 57 (54.3%) | 44 (93.6%) |
| Unknown | 31 (29.5%) | 1 (2.1%) |
| Number of tumor lesions |  |  |
| Multiple | 25 (23.8%) | 25 (53.2%) |
| Single | 47 (44.8%) | 22 (46.8%) |
| Unknown | 33 (31.4%) |  |
| History of NMIBC |  |  |
| No | 51 (48.6%) | 31 (66.0%) |
| Yes | 54 (51.4%) | 16 (34.0%) |
| Tumor grade |  |  |
| Low grade | 29 (27.6%) | 10 (21.3%) |
| High grade | 71 (67.6%) | 36 (76.6%) |
| PUNLMP | 1 (1.0%) | 1 (2.1%) |
| Unknown | 4 (3.8%) |  |
| Tumor stage |  |  |
| Tis | 1 (1.0%) |  |
| Ta | 50 (47.6%) | 25 (53.2%) |
| T1 | 49 (46.7%) | 22 (46.8%) |
| Unknown | 5 (4.7%) |  |
| Clinically risk stage |  |  |
| Low-risk | 17 (16.2%) | 3 (6.4%) |
| Intermediate-risk | 12 (11.4%) | 8 (17.0%) |
| High-risk | 40 (38.1%) | 36 (76.6%) |
| Unknown | 36 (34.3%) |  |

**Table S1.** Clinical features of all the recruited NMIBC patients.

**Table S2.** Clinical utility assessment of the utLIFE-UC assay.

| Recurrence monitoring cohort (47 patients at a 34% recurrence rate) | utLIFE-UC assessment before initial post-TURBT cystoscopy | Longitudinal utLIFE-UC assessment |
| --- | --- | --- |
| Estimated number of avoided cystoscopies (true negatives, TN) | 25 | 19 |
| Missed tumors (false negatives, FN) | 1 | 0 |
| Correct diagnosis of cancer (true positives, TP) | 15 | 16 |
| Per 1000 patients at a 20% recurrence rate | utLIFE-UC assessment before initial post-TURBT cystoscopy | Longitudinal utLIFE-UC assessment |
| Estimated number of avoided cystoscopies (TN) | 645 | 490 |
| Missed tumors (FN) | 12 | 0 |
| Correct diagnosis of cancer (TP) | 188 | 200 |
